# A prospective analysis of concentration of 25-OHD between northern and southern district in Japan in year-round study

**DOI:** 10.1097/MD.0000000000031340

**Published:** 2022-10-28

**Authors:** Akira Horikawa, Yuji Kasukawa, Michio Hongo, Hiroyuki Kodama, Akihisa Sano, Naohisa Miyakoshi

**Affiliations:** a Shizuoka Tokusyukai Hospital, Shizuoka, Japan; b Department of Orthopedic Surgery, Akita University Graduate School of Medicine, Akita, Japan; c South Akita Orthopedic Clinic, Seiwakai, Katagami, Japan.

**Keywords:** 25-OHD, BMD, eGFR

## Abstract

Although osteoporotic patients have already been recognized as having a low-volume vitamin D status, the concentration of active vitamin D precursor has not been studied in detail. This trial aimed to clarify the concentration of 25-hydroxyvitamin D (25-OHD), which is a natural type of vitamin D and compare between 2 separate areas in Japan. To compare and clarify the concentration of 25-OHD between 2 separate areas, Japanese patients who were diagnosed as having osteoporosis based on bone mineral density were studied. We analyzed 2 different hospitals’ patients whose residence is separated into a northern district (Akita city: north latitude 39” 43’) and a southern district (Shizuoka city: north latitude 34” 58’). Both of them have completely different daylight hours. Three-hundred sixty eight patients (174 in Akita, 194 in Shizuoka) were enrolled in this trial to compare the differences of concentration of 25-OHD by Welch’s t *t*-test. There were significant differences in the concentration of 25-OHD and age between them. Akita patients were significantly higher than that of Shizuoka patients despite Shizuoka having much daylight hours of Akita. In conclusion, there might be no relationship between the concentration of 25 OHD and exposure to sunlight.

## 1. Introduction

A low level of 25-hydroxyvitamin D (25OHD) has been reported in different countries^[1]^ although it is estimated to play an important role in skeletal growth^[2]^ and development. The synthesis of 25OHD has mainly 2 main pathways: intake from the diet^[3]^ and synthesis in the skin upon exposure to ultra violet rays.^[4–6]^ Moreover, it is widely acknowledged that 25OHD was an important parameter for determining osteoporotic status similar to bone mineral density and the risk of the fracture throughout life.^[7–10]^ However, the examination of this concentration has little performed due to certain medical restrictions in Japan.^[11]^

Recently, measuring the concentration of 25OHD has been approved by the Japanese Ministry of Health. Although there are some reports about the concentration of 25OHD fluctuating by season and differences in daylight hours,^[12]^ there are no reports comparing the concentration of 25OHD in different districts in Japan through the year.

As a consequence, we aimed to compare the concentration of 25OHD in 2 separate regions in Japan focusing on whether latitude and daylight hours are linked with the concentration of 25OHD.

## 2. Patients and methods

### 2.1. Study design and participants

From October 2018 to September in 2019, patients who received osteoporotic treatment as outpatients were enrolled in this trial in both Akita and Shizuoka city. They were diagnosed with osteoporosis if their bone mineral density values for the femoral neck, lumbar spine or radius were less than -2.5 standard deviation below the reference values. They had all previously received osteoporotic treatment or started intensive treatment for osteoporosis and were not taking any other kinds of medication. Additionally, none of them has taken medication for natural vitamin D preparation.

The number of each region’s patients were 174 in Akita (male 1, female 173), 194 in Shizuoka (male 3, female 191) and they had no severe dementia, Alzheimer’s, Parkinson’s disease or osteoporotic fracture who are forced to stay indoor activities. All of them had no diabetes mellitus or hyperparathyroidism which has linked to calcium metabolism and all had the daily habit of walking around 20 to 30 minutes during the daytime.

### 2.2. Measurements of laboratory finding

25OHD was assayed by a contract clinical laboratory (BML, Tokyo, Japan) and examined using the electrochemiluminescence immunoassay (ECLIA) kit. Moreover, data on the region, age, body mass index (BMI), serum calcium (Ca) concentrations, and estimated glomerular filtration (eGFR) also were collected from medical records.

All procedures performed in studies involving human participants were in accordance with the ethical standards of the 1964 Helsinki declaration, written and oral informed consent from all of the patients was obtained before the intervention of this trial, and the medical ethics committee of Akita University Graduate School of Medicine approved this study (approval number 1970).

### 2.3. Measurement of daylight hours

We researched and analyzed the daylight hours from October 2018 to September in 2019 both Akita and Shizuoka city via the Japanese metrological agency database (www.jma.go,jp).

### 2.4. Statistical analysis

Statistical analysis was performed using Microsoft Office Excel and the Statcel 3 program (OMS, Inc., Hyogo, Japan) after confirming all data’s adequacy by Cronbach’s alpha (over 0.8).

All data is presented as means and standard deviation. Both 25OHD, eGFR, serum Ca, their age, and lifestyle habits were analyzed by Welch’s *t*-test to compare differences between the groups. The relationship between age and the concentration of 25OHD was analyzed by simple regression analysis as to whether it is linearly associated with age or not.

## 3. Results

There was no significant difference in Ca and eGFR between these regions, although the Akita region patients’ age was significantly older than that of the Shizuoka region (Table 1). When it comes to most of their social economic status was middle class who can afford to buy sufficient diet and utilize for health care such as day-care (Table 2).

**Table 1:** Baseline characteristics of the two groups.

	Akita group N = 174	Shizuoka group N = 194	*P*-value
Age (y)	78.7 ± 3.3	75.7 ± 11.4	< 0.001^*^
BMI (kg/m^2^)	26.5 ± 5.3	25.9 ± 4.8	NS ^*^
BMD forearm (g/cm^2^)	0.425 ± 0.013	0.441 ± 0.039	NS ^*^
Serum calcium (g/dl)	9.4 ± 0.1	9.3 ± 0.3	NS ^*^
eGFR (ml/min/1.73 m^2^)	66.5 ± 13.2	64.8 ± 12.3	NS ^*^
25OHD (ng/ml)	17.1 ± 7.5	13.0 ± 5.9	< 0.001^*^
Total daylight hours	1849.9	2134.8	
Latitude (northern)	39° 43’	34° 58’	
Outdoor activity walking			
0–10 min/day	38	41	NS^*^
10–20 min/day	66	70	NS^*^
Over 20 min/day	70	83	NS^*^
Gardening			
Under 1 time/week	80	70	NS^*^
1–2 times/week	48	60	NS ^*^
Over 2 times/week	46	64	NS ^*^
Sun bathing			
Under 1 time/week	110	130	NS^*^
1–2 times/week	40	38	NS^*^
Over 2 times/week	24	26	NS^*^
Diet Salmon			
Under 1 time/week	18	55	NS^*^
1–2 times/week	53	100	NS^*^
Over 2 times/week	103	39	<0.05^*^
Whitebait			
Under 1 time/week	80	95	NS^*^
1–2 times/week	50	68	NS^*^
Over 2 times/week	44	31	NS^*^

Values are expressed as means and standard deviation.

Total daylight hours = total sum of daylight hours during year-round.

BMI = body mass index, BMD = bone mineral density, eGFR = estimated glomerular filtration.

* Welch’s *t*-test.

**Table 2:** Baseline characteristics of the two groups.

	Akita group	Shizuoka group	*p*-value
Alcohol			
Under 1 time/week	140	158	NS^*^
1–2 times/week	20	21	NS^*^
Over 2 times/week	14	15	NS^*^
Coffee			
Under 1 cup/day	153	174	NS^*^
1–2 cup/day	12	14	NS^*^
Over 2 cup/day	9	6	NS^*^
Smoking			
None	170	189	NS^*^
Under 20 cigarettes/day	4	5	NS^*^
Social economic status			
Low	20	30	NS^*^
Middle	140	135	NS^*^
High	14	29	NS^*^

Values are expressed as means and standard deviation.

* Welch’s *t*-tests

The concentration of 25OHD in Akita city was significantly higher than that in Shizuoka city (*P* < .001), although the total sum of daylight hours in Akita city was less than that in Shizuoka without accompanying sun skin damage by questionnaire. There was one significant change about the lifestyle habit for taking diet of Salmon between these regions. (Table 1,2).

**Figure 1.**
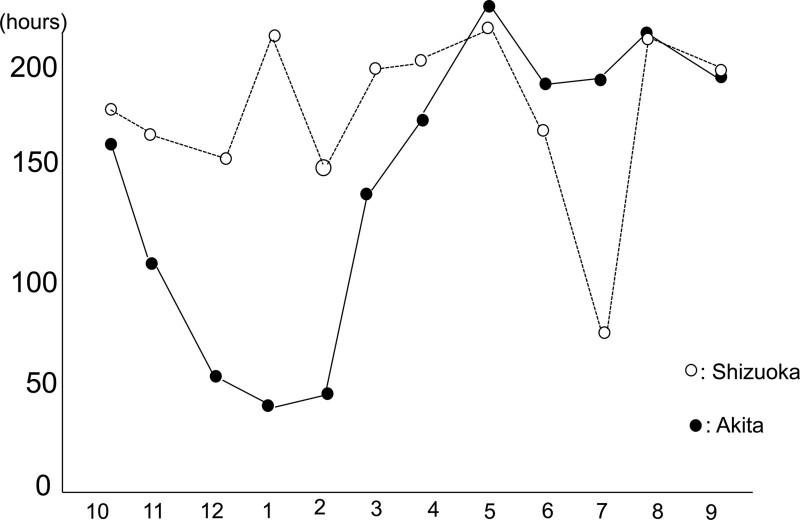
Total daylight hours by months. Total sum of daylight hours in Akita city was less than that in Shizuoka.

**Figure 2.**
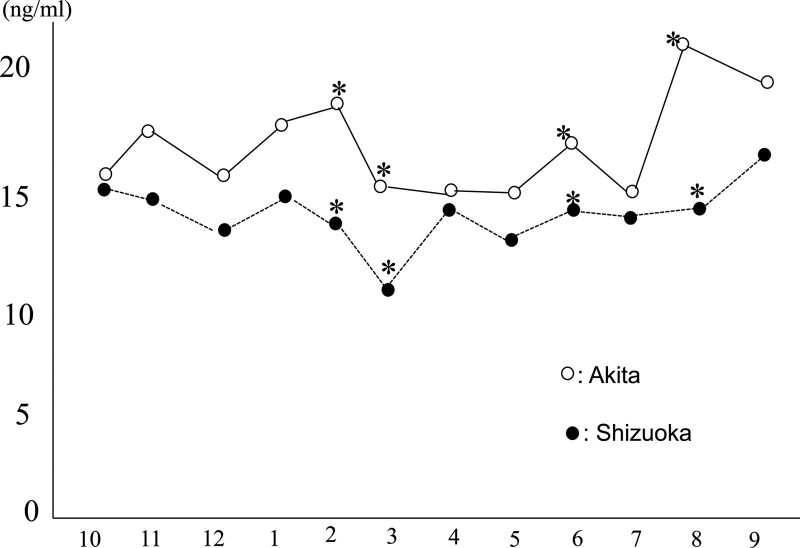
The concentration of 25OHD by months. The concentration of 25OHD in Akita city was significantly higher than that in Shizuoka city. 25-OHD = 25-hydroxyvitamin D.

The concentration of 25OHD per month was also described as follows in Figure 2. Furthermore, we examined the value for daylight hours in detail in Figure 1. The daylight hours chart indicated that the length of daylight hours in Shizuoka city is longer than that in Akita city especially from December to February except during rainy season from May to July. While the concentration of 25OHD in Shizuoka city was lower than that of Akita city in every month, there were especially significant differences in February, March, June and August (*P* = .048 and *P* < .001, respectively), Its results may reflect the half-time of the concentration of 25OHD is around 2 weeks that indicate the synthesis of 25OHD by daylight

The change of concentration of 25OHD in Shizuoka was relatively small although in Akita it was unstable and there is no relationship between the concentration of 25OHD and age in our analysis (Fig. 2).

**Figure 3.**
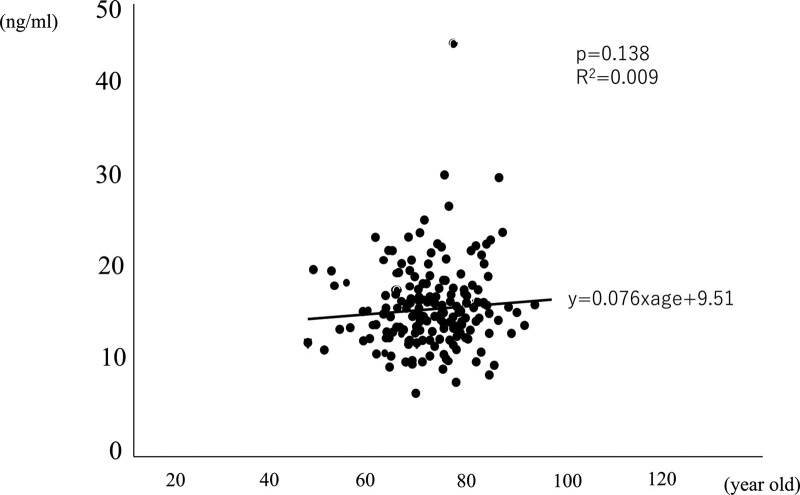
Correlation between age and 25OHD. There is no relationship between the concentration of 25OHD and age by single regression analysis. 25-OHD = 25-hydroxyvitamin D.

There is no relationship between the concentration of 25OHD and age by single regression analysis (Fig. 3).

## 4. Discussion

It has been widely recognized that biologically activate vitamin D controls the concentration of serum calcium by absorption from the intestine and by restricting of the secretion of parathyroid hormone,^[13]^ and the active hormone was derived from 25OHD. Moreover this biosynthesis is mainly divided 2 pathways: nutrition, and cutaneous synthesis caused by exposure to sunlight.^[14]^ The vitamin D status was defined by its concentration as follows; sufficient: serum 25OHD > 30 ng/ml, insufficient: 30 ng/ml > 25OHD > 20 ng/ml, and deficient: 20 ng/ml > 25OHD.^[15,16]^ The references values for 25OHD levels in Japan were reported as 14.6 or 16.5 ng/ml.^[13,17]^ Therefore, many osteoporotic patients were recommended to increase sunlight exposure. However, there is a discrepancy between the concentration of 25OHD and daylight hours in some global research.^[18]^ Although these results suggest that the concentration of 25OHD was not associated with total sum of sunlight, there were few reports about the relationship between these data in our country^[19]^ except for one child-care report.^[20]^ Our results indicated that Shizuoka city patients are relatively young and there is a high possibility for outdoor activity. It also shows that there is no inferiority about eGFR or in the concentration of calcium compared with Akita city patients, whereas the concentration of 25OHD was significantly lower in Shizuoka patients than that in Akita city patients. Surprisingly, there are many reports about the relevance between latitude and the concentration of 25OHD all over the world and they suggest that 25OHD in low latitude areas such as equatorial regions was not necessarily higher than that of high latitude areas such as the north European region.^[21–23]^ In general, the amount of production of 25OHD by sunlight was independent from latitude^[24]^ and skin pigmentation.^[25]^ That means those who live at higher latitudes and have more melanin require more sunlight-exposure compared to lower latitude dwellers with less melanin skin. Moreover, some reports indicated that about 80% of 25OHD was biosynthesized by sunlight.^[4,5]^ Contrary to our hypothesis that 25OHD in Shizuoka city would have a higher concentration of 25OHD than Akita city due to the lower latitude, similar genetics and more daylight hours, our results indicated a higher concentration of 25OHD in Akita. Besides, both regions are costal areas that seems to be similar in their patients’ dietary customs. Considering that focus was on the Akita regions, those patients were estimated to be ingesting large amounts of 25OHD by salmon in winter and it would be related to higher concentration of 25OHD especially in winter. There are some reports about high concentration of 25OHD in Japanese Niitaga prefecture residents who eat salmon in winter^[19]^ and some arctic northern Alaska people who have high 25OHD because of consuming large amounts of raw seals liver in winter.^[26]^ In addition, daylight hours in Akita is longer than that of Shizuoka during rainy season, these results reflect the concentration of 25OHD in Akita has significantly higher than that of Shizuoka because this season in Akita was relatively comfortable for outdoor activity such as walking in noon due to low temperature. These factors might be related to cause the differences in 25OHD between Akita and Shizuoka in rainy season and winter including other seasons.

Next, Akita city patients was significantly older than Shizuoka city patients which suggest the concentration of 25OHD was linearly associated with age.^[27,28]^ Whereas there is no evidence that the concentration of 25OHD is linearly associated with age in our analysis (Fig. 2) which were seen same results in several study.^[29,30]^

This result deny the reason Akita patients higher age is correlated with their higher concentration of 25OHD than Shizuoka patients.

There was a limitation that we did not find other reason why regional differences and daylight hours had no relevance to the concentration of 25OHD in this study that imply the participants’ effective exposure to light was also not evaluated although we confirmed the habit of daily walking during daytime around 20 to 30 minutes.

Finally, it is important for not only Japanese but all-over the world people to investigate the concentration of 25OHD and seek for the adequate biosynthesis of vitamin D status in the near future.

## 5. Conclusion

We investigated the osteoporotic patients’ relationship between the concentration of vitamin D status and daylight hours in separate district area in Japan. The concentration of 25OHD in high latitude area patients were significantly higher than that in low latitude area although low latitude area had longer daylight hours.

## Acknowledgments

The authors also would like to thank South Akita Orthopedic Clinic, Igarashi Memorial Hospital and Shizuoka Tokusyukai Hospital supported this study.

## Author contributions

**Data curation:** Hiroyuki Kodama, Akihisa Sano.

**Formal analysis:** Michio Hongo.

**Methodology:** Yuji Kasukawa.

**Supervision:** Naohisa Miyakoshi.

**Writing – original draft:** Akira Horikawa.
